# Changes in stiffness of the specific regions of knee extensor mechanism after static stretching

**DOI:** 10.3389/fbioe.2022.958242

**Published:** 2022-08-15

**Authors:** Yuanchun Zhu, Yanan Feng, Fangchao Huang, Yapeng Li, Wenjing Wang, Xueqiang Wang, Xiangyang Cao, Zhijie Zhang

**Affiliations:** ^1^ Department of Sport Rehabilitation, Shanghai University of Sport, Shanghai, China; ^2^ Rehabilitation Therapy Center, Luoyang Orthopedic Hospital of Henan Province, Orthopedic Hospital of Henan Province, Luoyang, China

**Keywords:** stiffness, knee extensor mechanism, quadriceps, patellar tendon, static stretching, range of motion (ROM)

## Abstract

Decreased muscle stiffness could reduce musculotendinous injury risk in sports and rehabilitation settings. Static stretching (SS) has been used to increase the flexibility of muscles and reduce muscle stiffness, but the effects of SS on the stiffness of specific regions of the knee extensor mechanism are unclear. The quadriceps femoris and patellar tendon are essential components of the knee extensor mechanism and play an important role in knee motion. Therefore, we explored the acute and prolonged effects of SS on the stiffness of the quadriceps femoris and patellar tendon and knee flexion range of motion (ROM). Thirty healthy male subjects participated in the study. Three 60-s SS with 30-s intervals were conducted in right knee flexion with 30° hip extension. We measured the ROM and stiffness of the vastus medialis (VM), vastus lateralis (VL), and rectus femoris (RF) and the proximal-(PPT), middle-(MPT), and distal-(DPT) region stiffness of the patellar tendon before and immediately after SS intervention, or 5 and 10 min after SS. The stiffness of the quadriceps muscle and patellar tendon were measured using MyotonPRO, and the knee flexion ROM was evaluated using a medical goniometer. Our outcomes showed that the ROM was increased after SS intervention in all-time conditions (*p* < 0.01). Additionally, the results showed that the stiffness of RF (*p* < 0.01) and PPT (*p* = 0.03) were decreased immediately after SS intervention. These results suggested that SS intervention could be useful to increase knee flexion ROM and temporarily reduce the stiffness of specific regions of the knee extensor mechanism.

## Introduction

The knee extensor mechanism refers to the convergence of the four quadriceps muscles into the quadriceps tendon, which is attached to the proximal patella and extends distally to the tibial tuberosity through the patellar tendon (Dan et al., 2018). The muscle and tendon play a crucial role in the extension and flexion of the knee joint, which enables the efficient functioning of the knee extensor mechanism in stabilization and locomotion (Ostlere, 2013). Regarding muscle strain incidence, quadriceps femoris [especially the rectus femoris (RF)] is one of the most frequently affected (Cross et al., 2004; Orchard et al., 2020). Previous studies found that higher muscle–tendon unit stiffness could contribute to impaired function and decreased range of motion (ROM) (Watsford et al., 2010; Geertsen et al., 2015). Stiffness is one of the mechanical properties associated with the muscle and tendon adaptation process, representing the relationship between the force imposed on soft tissue and the deformation it applies (Bohm et al., 2015). To reduce muscle strain and improve joint ROM, it is crucial to reduce the stiffness of muscle and tendon (Watsford et al., 2010; Geertsen et al., 2015).

Static stretching (SS) is commonly utilized to increase muscle flexibility, decrease muscle stiffness, and reduce musculotendinous injury risk in sports and rehabilitation settings (Malliaropoulos et al., 2004; Morse et al., 2008; Konrad et al., 2017). A systematic review revealed that the four factors related to the impact of stretching on flexibility are intervention frequency, duration, position, and intensity (Apostolopoulos et al., 2015). Flexibility is usually assessed by measuring the ROM. Recently, Nakamura et al. reported the acute effect of 180-s of three different SS intensities on the ROM and the passive stiffness of quadriceps muscles, with results showing that the ROM was increased after 100% and 120% (high) intensities of SS intervention and only the RF stiffness was reduced significantly after 100% intensity SS (Nakamura et al., 2020). However, this study has a limitation: only acute effects were investigated. Past studies reported that SS acutely reduces the stiffness of gastrocnemius (Konrad et al., 2019) and hamstrings (Hatano et al., 2019). Hatano et al. (2019) reported that hamstrings stiffness decreased significantly 20 min after SS, and knee ROM increased significantly 30 min after SS. A limited number of research studies have explored the acute effect of SS on quadriceps stiffness and knee flexion ROM (Nakamura et al., 2020; Takeuchi et al., 2021). Nevertheless, none of the studies examined the chronic effects of SS on quadriceps stiffness and knee flexion ROM.

In addition, muscular function and integrity are close-knit and are related to variations in the mechanical properties of tendons (McCrum et al., 2018). Some researchers reported that tendon stiffness reduced after 300 s of SS (Kubo et al., 2002; Burgess et al., 2009). However, Kay et al. found that 3 min of SS decreased muscle stiffness but there was no change in Achilles tendon stiffness (Kay and Blazevich, 2009). Thus, the effects of SS interventions on tendon properties remain unclear. Moreover, to the best of our knowledge, no study has explored the acute and prolonged effects of SS on specific regions of patellar tendon stiffness. Thus, the purpose of our study was to investigate 1) the acute and prolonged effects of SS intervention on the knee flexion ROM and 2) the acute and prolonged effects of SS intervention on the stiffness of the proximal (PPT), middle (MPT), and distal (DPT) regions of the patellar tendon and the vastus medialis (VM), vastus lateralis (VL), and RF. We hypothesized that there would be a significant decrease in RF stiffness and a significant increase in knee ROM, both of which would persist for all testing periods after stretching. Furthermore, we hypothesized that the stiffness of the patellar tendon does not change before and after stretching. We believed that significant ROM and RF stiffness changes after SS might reduce the risk of knee extensor mechanism injuries such as RF strain in physical therapy and rehabilitation practice.

## Materials and methods

### Experimental design

A repeated measures experimental design was conducted to examine the acute and prolonged impacts of SS protocol (100% intensity and 3 min duration) on ROM, stiffness of the quadriceps (VM, VL, and RF), and patellar tendon (PPT, MPT, and DPT) in the lead extremity (ball kicking preference). Our SS intensity and duration option refers to a previous study (Nakamura et al., 2020). The dominant extremity of all participants was the right leg. In this study, the ROM and stiffness of the quadriceps muscles and patellar tendon were examined before (PRE) and immediately after SS (POST), or 5 (POST 5) and 10 (POST 10) min after SS. As for this measurement sequence, we measured the knee flexion ROM first and then assessed the stiffness.

### Participants

Thirty healthy male volunteers (21.5 ± 1.2 years, 176.5 ± 5.2 cm, and 70.4 ± 6.3 kg) were recruited for this experiment. Participants who regularly performed strength and flexibility training or had a history of neuromuscular diseases and lower limb musculoskeletal system injuries were excluded. All volunteers were requested not to conduct flexibility or resistance training of the lower extremities for the duration of the experiment. All volunteers signed written informed consent to participate in the present study. This research was approved by the Ethics Committee of the Luoyang Orthopedic Hospital of Henan Province (KY 2020-003-02), China, and conformed to the Declaration of Helsinki.

### Knee flexion ROM

**FIGURE 1:**
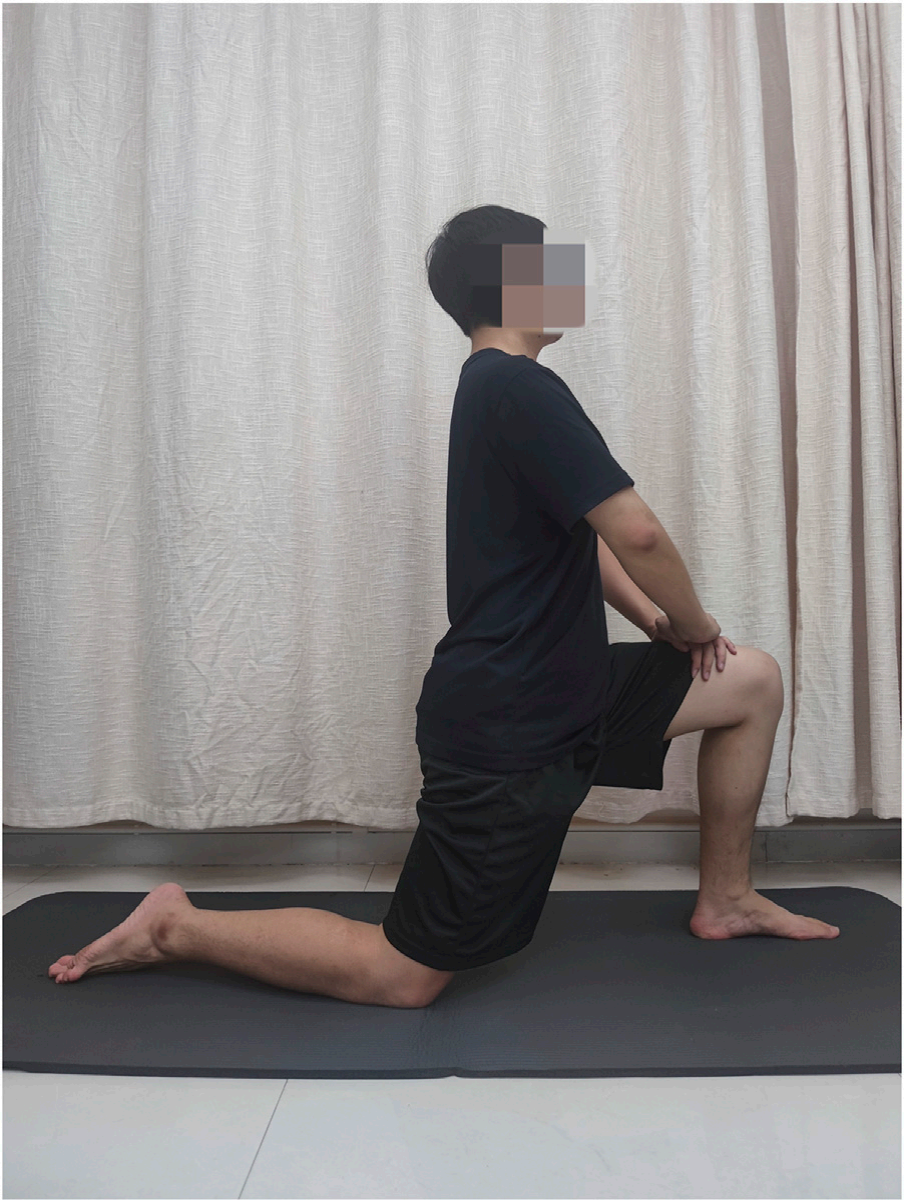
Reference extremity posture of static stretching (SS).

The methodology for assessing ROM was the same as that of the previous study (Takeuchi et al., 2021). Subjects were positioned with the left hip and knee flexed at 90° and the right limb with the knee flexed and the hip extended at 30° as the reference extremity posture (Figure 1). Then, the researcher flexed the knee slowly and passively from the reference extremity posture to the angle just before the volunteers began to feel pain or discomfort (Nakamura et al., 2020). We used a medical goniometer to measure the ROM twice and the mean value for the subsequent analysis.

### Equipment

The MyotonPRO (Myoton AS, Tallinn, Estonia) is a non-invasive portable machine utilized to quantify tendon and muscle stiffness in this study. The device works by exerting a small mechanical impact on the tissue of interest area, perpendicular to the skin surfaces. The probe’s tip is then pushed to the measured site to reach the desired depth. After the red light turns green, the machine automatically implements five short pulses (0.8 s between taps) to cause mechanical oscillations of the tissue. According to the previous study, we found good intra- and inter-tester reliability for measuring the stiffness of the quadricep muscles and patellar tendon using the MyotonPRO (Chen et al., 2019). MyotonPRO recorded the parameters of stiffness. Stiffness was described as newtons/meter (N/m). The higher the value, the stiffer the soft tissue.

### Stiffness measurements of quadriceps muscle and patellar tendon

We measured the stiffness of the VM, VL, RF, PPT, MPT, and DPT using MyotonPRO. All subjects were tested in the same physical therapy room, and measurements were performed at the ambient temperature of 25°C in a relaxed position with knee and hip flexed at 90°. The stiffness of the RF at the reproducible location is about two-thirds of the distance between the anterior superior iliac spine and the patella apex (Agyapong-Badu et al., 2016). In order to locate the VM and VL’s muscular abdomen more accurately, participants were asked to actively extend their hip and knee joints. The most prominent site of each muscle belly was used as a measurement point (Chen et al., 2019). The stiffness of the patellar tendon was measured at defined anatomic areas (PPT, the patella apex as a superior landmark; MPT, the middle of the patellar tendon between the patella apex and tibial insertion; and DPT, tibial insertion as a distal landmark) (Dickson et al., 2020). All testing sites were marked on the skin by Yuanchun Zhu using a non-toxic pen. We measured the stiffness of each position five times and used the average value for analysis.

Before the SS intervention maneuver, we randomly examined the test–retest reliability of stiffness measurement for the quadriceps femoris and patellar tendon using ten limbs in ten healthy male subjects.

### Static stretching

The SS maneuver was conducted similar to the ROM measurement. The 100% intensity of SS intervention was determined by the ROM in the PRE value just before the volunteers began to feel pain or discomfort (Nakamura et al., 2020). Three 60-s SS with 30-s intervals were conducted with the same knee flexion angle. Subjects were required to remain relaxed and hold their trunk upright during SS duration. During the rest period of each SS, the subjects lay on the treatment couch in the supine position and remained completely relaxed.

### Statistical analyses

All descriptive data are presented as means ± standard deviation. The statistical analyses were conducted using SPSS (version 26.0, IBM, United States). The Shapiro–Wilk test was utilized to examine the normality of all data. Measurement test–retest reliability was analyzed using the intraclass correlation coefficient and coefficient variation. We executed a one-way repeated measure analysis of variance (ANOVA) to assess time effects (PRE vs POST vs POST 5 vs POST 10) for the ROM. For the stiffness, a two-way repeated measure ANOVA was utilized to evaluate [time (PRE vs POST vs POST 5 vs POST 10) and regions (RF vs VM vs VL vs PPT vs MPT vs DPT)] and analyze the main and interaction effect. Post-hoc analyses using Bonferroni multiple comparison tests were performed if a significant interaction effect was detected. Statistical significance was considered as *p* values <0.05.

## Results

### Reliability of the stiffness measurements

Good test–retest reliability (ICC, CV) was found for the stiffness measurements of the quadriceps and patellar tendon (Table 1).

**TABLE 1:** Reliability assessment of stiffness measurements (Mean ± SD).

	Test	Retest	ICC (95%CI)	CV (%)
RF (N/m)	268.3 ± 34.3	268.4 ± 34.0	0.950 (0.810–0.987)	12.4
VM (N/m)	292.3 ± 34.0	288.7 ± 37.3	0.985 (0.923–0.996)	12.0
VL (N/m)	385.6 ± 56.4	382.4 ± 58.4	0.993 (0.971–0.998)	14.6
PPT (N/m)	821.1 ± 117.1	821.8 ± 126.4	0.983 (0.933–0.996)	14.4
MPT (N/m)	620.2 ± 105.7	622.7 ± 106.2	0.991 (0.966–0.998)	16.6
DPT (N/m)	714.5 ± 123.7	723.6 ± 131.4	0.979 (0.924–0.995)	17.3

RF, rectus femoris; VM, vastus medialis; VL, vastus lateralis; PPT, proximal patellar tendon; MPT, middle patellar tendon; DPT, distal patellar tendon; ICC, intraclass correlation coefficient; CV, coefficient variation.

### Changes in the knee flexion ROM

**FIGURE 2:**
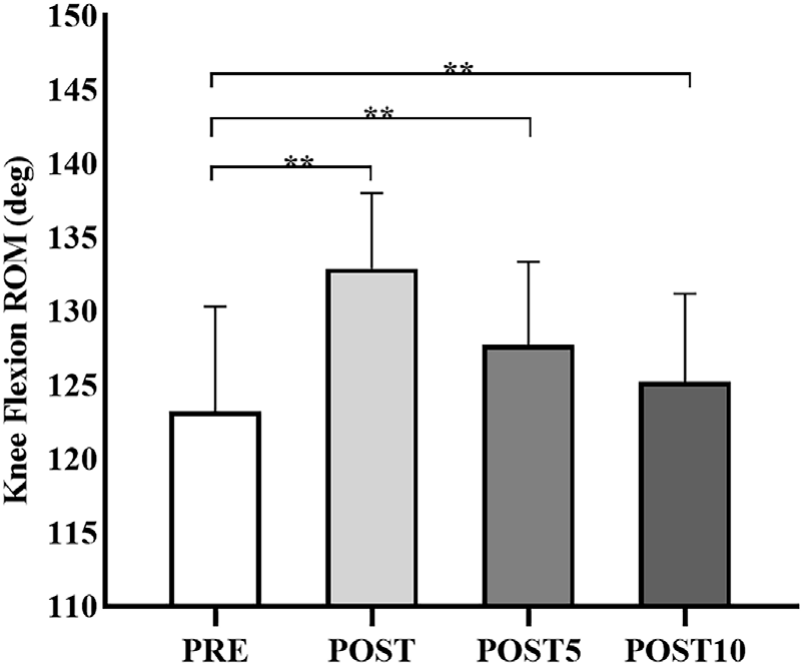
Knee flexion range of motion (ROM) changes before (PRE) and immediately after static stretching (POST), or 5 (POST 5) and 10 (POST 10) min after static stretching. ^∗∗^ *p* < 0.01.

The changes in the ROM before and after SS are shown in Table 2 and Figure 2. The ROM after SS in all-time conditions was greater than that before SS intervention (*p* < 0.01). There was a significant increase in knee flexion ROM after the SS intervention (*p* < 0.01). In addition, the post-hoc test revealed that the knee flexion ROM was significantly higher in POST and POST 5 than in POST 10 (*p* < 0.01).

**TABLE 2:** Changes in RF, VM, VL, PPT, MPT, and DPT stiffness and knee flexion ROM before and after the static stretching intervention (Mean ± SD).

	PRE	POST	POST 5	POST 10
RF (N/m)	274.7 ± 39.1	258.4 ± 38.2^∗∗^	266.4 ± 38.7^##^	270.0 ± 37.4^##^
VM (N/m)	295.4 ± 43.8	292.1 ± 40.9	293.9 ± 39.7	298.3 ± 40.5
VL (N/m)	366.2 ± 51.2	363.5 ± 48.4	364.4 ± 51.2	367.1 ± 50.7
PPT (N/m)	858.3 ± 92.2	831.0 ± 90.9^∗^	849.4 ± 100.7	852.7 ± 96.2^#^
MPT (N/m)	635.7 ± 109.7	641.4 ± 113.1	646.9 ± 116.3	642.2 ± 107.2
DPT (N/m)	744.1 ± 114.2	754.5 ± 105.7	755.2 ± 104.3	753.1 ± 104.5
ROM (°)	123.2 ± 7.1	132.9 ± 5.1^∗∗^	127.7 ± 5.6^∗∗,##^	125.2 ± 5.9^∗∗,##,†^

PRE, before the SS; POST, immediately after the SS; POST 5, 5 min after the SS; POST 10, 10 min after the SS; RF, rectus femoris; VM, vastus medialis; VL, vastus lateralis; PPT, proximal patellar tendon; MPT, middle patellar tendon; DPT, distal patellar tendon; ROM, range of motion.

∗p < 0.05; significant difference with PRE.

∗∗*p* < 0.01; significant difference with PRE.

#*p* < 0.05; significant difference with POST.

##*p* < 0.01; significant difference with POST.

† *p* < 0.01; significant difference with POST 5.

### Changes in the stiffness

**FIGURE 3:**
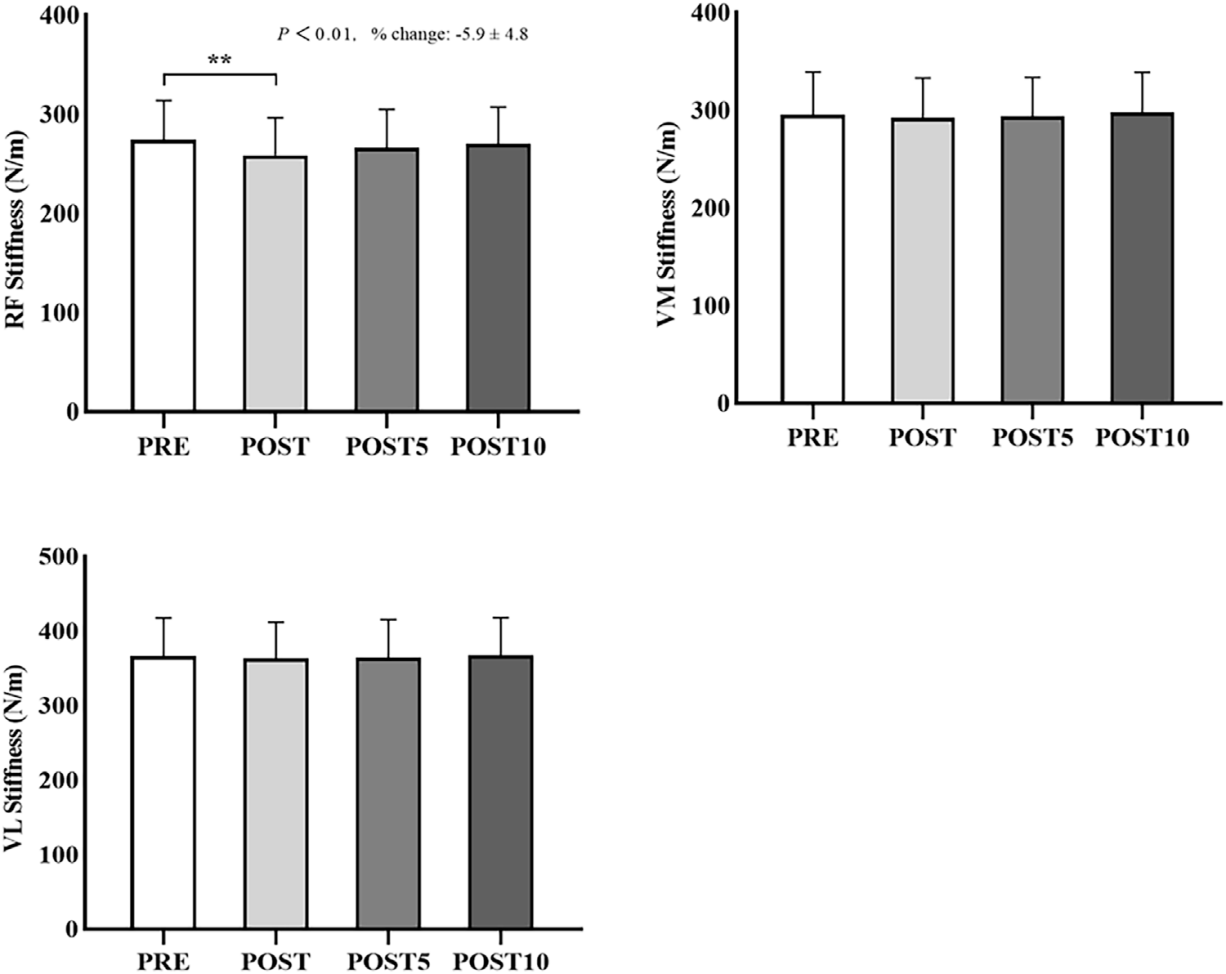
Stiffness changes of the rectus femoris (RF), vastus medialis (VM), and vastus lateralis (VL) muscles before (PRE) and immediately after static stretching (POST), or 5 (POST 5) and 10 (POST 10) min after static stretching. ∗∗*p* < 0.01.

**FIGURE 4:**
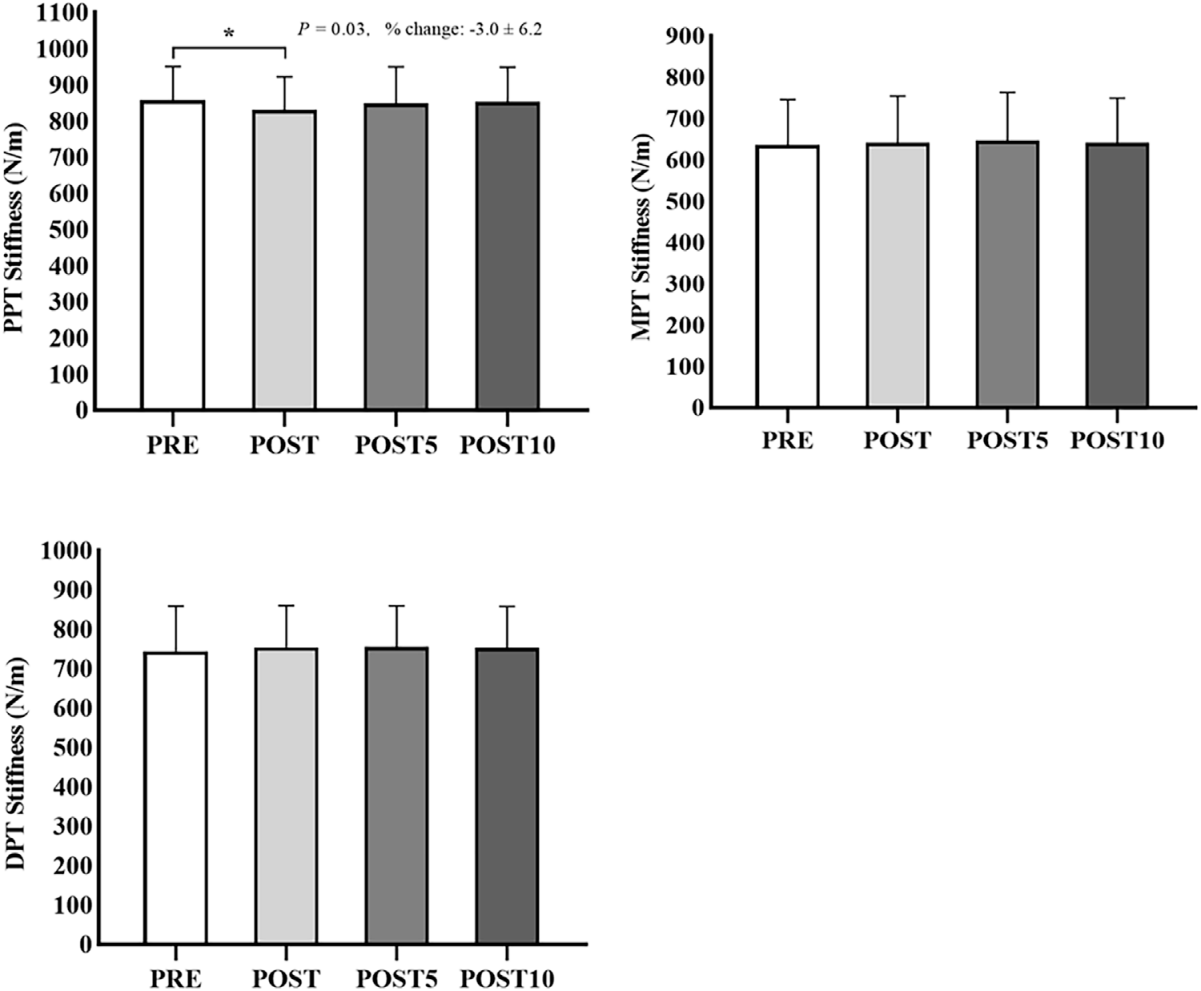
Stiffness changes of the proximal (PPT), middle (MPT), and distal (DPT) regions of the patellar tendon before (PRE) and immediately after static stretching (POST), or 5 (POST 5) and 10 (POST 10) min after static stretching. ^∗^ *p* < 0.05.

The stiffness changes of the patellar tendon and quadriceps muscles before and after SS are shown in Table 2 and Figures 3 and 4. The two-way repeated ANOVA revealed a significant interaction effect (*p* = 0.02, F = 2.00, η_p_^2^ = 0.05) and the significant time effect in the stiffness of RF and PPT (*p* < 0.01, F = 16.93, η_p_^2^ = 0.65; *p* = 0.03, F = 3.87, η_p_^2^ = 0.30, respectively). When comparing time points between PRE and POST in the SS intervention, the Bonferroni’s test revealed a significant decrease in the stiffness of RF and PPT (*p* < 0.01, 95% confidence interval, 9.0–23.6; *p* = 0.03, 95% confidence interval, 2.0–52.7), whereas no significant differences were found in POST 5 and POST 10 compared with PRE stiffness. In addition, there was no significant change in VM, VL, MPT, and DPT stiffness after the SS intervention compared with PRE stiffness.

## Discussion

The present research study explored the acute and prolonged effects of SS intervention on the ROM and VM, VL, and RF muscle stiffness and PPT, MPT, and DPT tendon stiffness. Accordingly, the major findings of our study were that 1) the SS intervention increased the knee flexion ROM, and this change was continued till 10 min after SS; and 2) the RF stiffness and PPT stiffness significantly decreased after the SS, whereas this reduction was not sustained till 5 min after SS. Although some studies have investigated the effects of SS intervention on the gastrocnemius (Nakamura et al., 2011; Sato et al., 2020), hamstring (Hatano et al., 2019; Fukaya et al., 2021), and the quadriceps femoris (Nakamura et al., 2020; Takeuchi et al., 2021). Moreover, this is the first study to investigate the acute and prolonged effects of SS intervention on ROM and specific regions of stiffness of the patellar tendon and quadriceps muscle.

According to the study results, knee flexion ROM increased for 10 or more minutes after 100% intensity SS intervention. This acute effect was in alignment with the previous research (Nakamura et al., 2020), which revealed that 100% and 120% intensities of SS intervention immediately increased the knee flexion ROM. Kataura et al. (2017) found that high-intensity SS is more effective for decreasing stiffness and increasing ROM than low-intensity SS. Additionally, recent research explored the influences of SS with low intensity and long duration (50% and 240 s) or high intensity and short duration (120% and 100 s) on ankle dorsiflexion ROM, and results revealed that the ROM was significantly higher at (120% and 100 s) than that of (50% and 240 s) conditions after SS intervention (Fukaya et al., 2020). Therefore, the SS at greater intensity (100% intensity) performed in our study may reduce the SS duration required to decrease stiffness. A change in ROM after SS is related to changes in stretching tolerance (peak passive torque) and muscle stiffness (Behm et al., 2016). Recent studies have reported that the proposed mechanisms of ROM increase could be related to increased stretch tolerance modulation and the stretching sensation of the participants (Kay et al., 2015; Freitas et al., 2018). Previous studies have proposed increased stretch tolerance due to the reduction of the sensations of discomfort and pain accompanied by the modulation of neuropsychological factors after stretching (Folpp et al., 2006; Law et al., 2009). Similarly, Hatano et al. (2019) reported the chronic influence of 5 min of SS on hamstrings muscles and revealed that the increase in knee extension ROM lasted 30 or more minutes after SS intervention. Nevertheless, due to the differences in mechanical properties and muscle structure, the effects of SS on different muscles and joint ROM are still unclear and need to be investigated further, even when SS intervention of equal intensity and duration is performed.

The results of our study showed that RF muscle and PPT stiffness significantly decreased after SS, whereas there were no significant alterations in the VM, VL, MPT, and DPT stiffness. A previous study examined the effects of the 120% intensity with two different durations (1 and 3 min) and 110% intensity with 3-min duration SS intervention on RF muscle stiffness, and the authors reported a significant reduction in the 110% intensity SS group, but the stiffness of RF muscle was not changed in 120% intensity SS group (Takeuchi et al., 2021). These results revealed that the SS intensity is more crucial than duration in decreasing the RF stiffness (Takeuchi et al., 2021). Moreover, a recent study investigated the effects of three different SS intensities (80 vs 100 vs 120%) on the RF stiffness. Results showed that the RF stiffness decreased only after 100% intensity SS (Nakamura et al., 2020). Therefore, we used SS at 100% intensity for 3 min as our intervention parameters for this study. At 100% intensity, the RF stiffness was reduced significantly after the SS maneuver, with no changes in the stiffness of the VM and VL, which could be associated with the structural differences between the biarticular and monoarticular muscles. We used a flexed knee and extended hip stretch position, and the RF as a biarticular muscle may have been stretched more fully compared to the VM and VL muscles, resulting in the stiffness decrease. The stiffness changes after SS differed from what we hypothesized; according to the outcomes of the present study, the decreased RF stiffness recovered to baseline within 5 min. Assuming that the reduction in muscle stiffness is based on a stretch-induced increase in resting sarcomere length (Gajdosik, 2002), these results may be due to the impact of the sarcomere length recovered 5 min after SS. Therefore, the effect of SS interventions on the constitutive muscles of the quadriceps may be different. More investigation is needed on the influence of various types of stretching on the stiffness of each muscle that makes up the quadriceps.

Interestingly, the SS intervention only significantly decreased the PPT stiffness, whereas there were no significant alterations in the MPT and DPT stiffness. Decreased tendon stiffness after SS training was also reported by Kubo et al. (2002). Nakamura et al. (2013) reported that 5 min SS increased the tendon stiffness. However, other researchers found unchanged tendon stiffness after a single SS intervention (Kay and Blazevich, 2009; Kay et al., 2015). Indeed, the change in tendon stiffness induced by SS has been debated among investigators. One possible interpretation for the contradictory results of the effect of SS on changes in tendon stiffness could be the methodological differences (e.g., measurement area, stretching time, stretching method, and strength). Moreover, the patellar tendon architecture of humans is depicted as viscoelastic, which is sensitive to increased mechanical loading environment and adapts by altering its material, morphological, and mechanical properties (Kongsgaard et al., 2007; Seynnes et al., 2009). Indeed, a previous study has demonstrated that the prominent histopathological changes of the patellar tendon typically occur in the proximal region accompanied by reduced tendon stiffness (Wiesinger et al., 2020), which may present a deficit concerning muscle function (Pearson and McMahon, 2012; Pearson and Hussain, 2014). Previous studies have reported a possible increase in tendon displacement during stretching, resulting in decreased tendon stiffness (Kubo et al., 2002; Burgess et al., 2009). We also considered that the difference in stress applied to the patellar tendon during the SS intervention might decrease PPT stiffness. Nevertheless, the mechanism of this temporary decrease in stiffness in specific areas of the patellar tendon after SS remains unclear, and whether this change in stiffness is associated with pathological changes in the patellar tendon requires further study. Previous studies found that the higher muscle–tendon unit stiffness could contribute to impaired function and decreased ROM (Watsford et al., 2010; Geertsen et al., 2015). SS is commonly utilized to increase muscle flexibility, decrease muscle stiffness, and reduce musculotendinous injury risk in exercise and rehabilitation settings (Malliaropoulos et al., 2004; Konrad et al., 2017). Additionally, flexibility is usually assessed by measuring the ROM. Our study showed that knee ROM was significantly increased after SS, and stiffness of RF and PPT was significantly decreased after SS. This suggested that when we intervene with 3 min of 100% intensity SS in physical therapy and rehabilitation practice, it may reduce the risk of knee extensor mechanism injuries such as RF strains.

There were several limitations in this study. First, the subjects were not regular exercisers. Thus, it is necessary to investigate the influences of SS intervention in athletes who regularly perform exercise training. Second, we did not consider female subjects in the study, although it is well known that there are differences in structure and function between the sexes. Third, the SS intensity was determined by reference to ROM (Nakamura et al., 2020; Takeuchi et al., 2021). However, ROM is influenced by subjective factors of stretching tolerance. Fourth, we did not include a control group without SS intervention. Thus, future studies should investigate the effect of SS on the stiffness of specific regions of the knee extensor mechanism in gender-specific athletes and sedentary populations.

## Conclusion

The results showed that the SS intervention increased the knee flexion ROM, and this change was continued till 10 min after SS; the RF stiffness and PPT stiffness significantly decreased after the SS intervention, whereas this reduction was not sustained till 5 min after SS. It is suggested that SS intervention could be useful to increase ROM and temporarily reduce the stiffness of specific regions of the knee extensor mechanism. However, SS interventions with different stretching intensities and durations for further studies are necessary to investigate whether they effectively prevent injuries related to the quadriceps and patellar tendon.

## Data Availability

The original contributions presented in the study are included in the article/Supplementary Material; further inquiries can be directed to the corresponding author.
